# High-efficiency solar thermoelectric conversion enabled by movable charging of molten salts

**DOI:** 10.1038/s41598-020-77442-y

**Published:** 2020-11-24

**Authors:** Chao Chang, Zongyu Wang, Benwei Fu, Yulong Ji

**Affiliations:** 1 Institute of Marine Engineering and Thermal Science, Marine Engineering College, Dalian Maritime University, Dalian, 116026 People’s Republic of China; 2 State Key Laboratory of Metal Matrix Composites, School of Materials Science and Engineering, Shanghai Jiao Tong University, Shanghai, 200240 People’s Republic of China

**Keywords:** Energy harvesting, Devices for energy harvesting, Solar energy, Solar thermal energy

## Abstract

Solar energy as an abundant renewable resource has been investigated for many years. Solar thermoelectric conversion technology, which converts solar energy into thermal energy and then into electricity, has been developed and implemented in many important fields. The operation of solar–thermal–electric conversion systems, however, is strongly affected by the intermittency of solar radiation, which requires installation of thermal storage subsystems. In this work, we demonstrated a new solar–thermal–electric conversion system that consists of a thermoelectric converter and a rapidly charging thermal storage subsystem. A magnetic-responsive solar–thermal mesh was used as the movable charging source to convert incident concentrated sunlight into high-temperature heat, which can induce solid-to-liquid phase transition of molten salts. Driven by the external magnetic field, the solar–thermal mesh can move together with the receding solid–liquid interface thus rapidly storing the harvested solar–thermal energy within the molten salts. By connecting with a thermoelectric generator, the harvested solar–thermal energy can be further converted into electricity with a solar–thermal–electric energy conversion efficiency up to 2.56%, and the converted electrical energy can simultaneously light up more than 40 orange-colored LEDs. In addition to stable operation under sunlight, the charged thermal storage subsystem can release the stored heat and thus enables the solar–thermal–electric system to continuously generate electricity after removal of solar illumination.

With the continuous consumption of fossil fuels and the rising pressure on the environment, the development and utilization of renewable energy have received tremendous attention in the energy research field worldwide. Among them, solar energy has gained great interest due to the advantages such as high capacity, global distribution, sustainability, environmental friendliness^1–3^. Solar–thermal technology is regarded as the most efficient and direct way to harness solar energy. It has already been adopted in multiple fields such as domestic heating^4,5^, steam generation^6–8^, seawater desalination^9,10^ and solar–thermal power plants^11^. Among them, solar–thermal–electric conversion is recognized as one of the most promising technologies to convert solar energy into electricity and such technology has been implemented in many industrial fields^12–14^. Unlike photovoltaic systems, solar–thermal–electric conversion systems store solar energy as heat in thermal storage materials. Therefore, these systems do not need the installation of electrical batteries, which not only simplifies the structure of the system but also significantly reduces the associated cost.

In solar–thermal–electric conversion systems, thermoelectric materials that enable direct conversion between thermal energy and electrical energy through the Seebeck effect have been extensively investigated^15,16^. The thermoelectric energy conversion efficiency depends on the dimensionless figure of merit *ZT* = *S*^*2*^*σTk*^*−1*^, where S, *σ, T* and* k* are the Seebeck coefficient, electrical conductivity, average absolute temperature and thermal conductivity, respectively^17^. It can be seen that a high temperature is beneficial for achieving a large figure of merit for the thermoelectric materials. To improve the performance of the solar–thermal–electric systems, considerable efforts had been devoted to adding concentrators^18–20^, designing heat sink^21^ and optimizing the cooling section^22–24^. Although many novel solar–thermal–electric convertors have been designed and fabricated, continuous and stable solar–thermal–electric conversion is still challenging. The research on solar–thermal–electric systems with high-performance solar–thermal storage systems was rarely reported. In fact, solar energy is strongly affected by weather, season, day and night, which causes the discrepancy between energy demand and supply. In order to overcome the inherent shortcomings of solar radiation such as intermittency, low energy density and uneven distribution, solar–thermal–electric conversion systems require the installation of extra thermal storage subsystems. The thermal storage materials within these subsystems are charged during day time, and the charged thermal energy can be released to maintain normal operation of the solar–thermal–electric systems day and night^25–29^. Therefore, an efficient solar–thermal storage subsystem becomes an essential component for the high-performance solar–thermal–electric systems.

Thermal storage materials are mainly divided into sensible thermal storage materials and latent thermal storage materials. Compared to the sensible thermal storage materials, the latent thermal storage materials have larger heat storage capacity and narrower heat-releasing temperature range, which can greatly increase the heat storage capacity and reduce the volume of the storage system. Current latent thermal storage materials, however, generally have a low thermal conductivity, which severely limits the thermal storage efficiency of the system and hinders its practical applications. In the past, tremendous efforts have been devoted to enhancing the thermal conductivity of phase change materials through direct incorporation of high-thermal-conductivity materials such as carbon materials^30,31^, metal foam^32–34^ and nanoparticles^35–39^. These approaches are suitable for the organic phase change thermal storage materials systems at low temperatures, it is challenging to implement in molten salts at medium-to-high temperatures^40^. Recently, we reported a novel movable charging method by using a magnetically-responsive photothermal mesh, which doubles solar–thermal energy harvesting rates while maintaining storage capacity of high-temperature molten salt phase change materials^41^.

In this work, by taking the advantage of fast charging under the movable charging mode we demonstrated a novel solar–thermal–electric energy harvesting system containing a solar–thermal storage subsystem and a thermoelectric conversion subsystem. In the solar–thermal storage subsystem, high-temperature molten salts were used as the phase change thermal storage material. Magnetically-responsive solar–thermal conversion meshes (MSCMs) were used to absorb solar energy and charge the molten salts under a movable charging mode. The surface of MSCMs was wrapped by graphite and PDMS (polydimethylsiloxane), which achieved high solar absorption and good compatibility with the high-temperature molten salts. The MSCMs can efficiently convert solar energy into heat to melt the molten salts, and its porous structure allows the mesh to quickly pass through the melted salts under the attraction from the magnet placed beneath. The incident sunlight penetrated through the melted liquid molten salts to rapidly advance the solid–liquid charging interface, thereby realizing rapid movable charging. By connecting the charged solar–thermal storage subsystem to a thermoelectric generator, we demonstrated that the harvested solar–thermal energy within high-temperature molten salts could produce sufficient electrical power to light up multiple light-emitting diodes (LEDs). Compared to conventional static charging systems, the movable charging led to increased voltage output and prolonged duration of electricity generation after removal of solar illumination.

## Results and discussion

**Figure 1:**
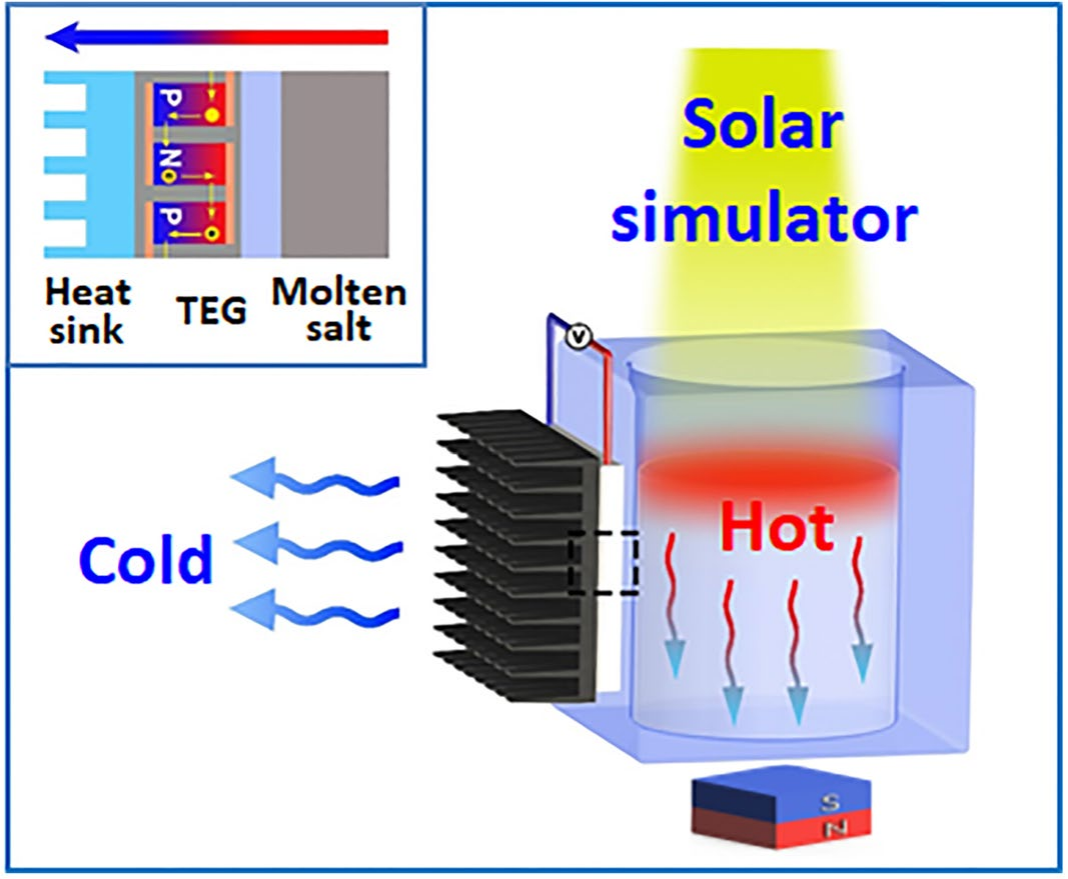
Schematic of the solar–thermal–electric conversion system consisting of a movable charging solar–thermal storage subsystem and a thermoelectric conversion subsystem. A magnet is placed at the bottom of the solar–thermal storage subsystem to achieve movable charging of the molten salts in the container.

The high storage temperature of the charged molten salts can generate a large temperature difference, thus enabling conversion of the stored high-capacity thermal energy into electricity through the Seebeck effect. As shown in Fig. 1, a thermoelectric chip is attached onto one side of an aluminum container, which was loaded with molten salts and was used as the hot side. The cold side of the generator was connected to a metallic fin and the fin was cooled by a fan. The MSCM was placed on top of the molten salts to convert solar energy into heat, and a magnet was placed at the bottom of the aluminum container. To minimize heat loss, except the sidewall connected to the heat sink, the bottom surface and the other three sidewalls of the solar–thermal storage subsystem were wrapped by thermal insulating glass fibers with a thickness of 1 cm. Upon solar irradiation, the MSCM immediately converts solar energy into heat and the converted heat is stored within the molten salts. Under the attraction from the magnet, the MSCM is able to pass through the melted liquid molten salts, and quickly advance the solid–liquid melting interface. The thermoelectric generator was activated to generate electrical power by the temperature difference between molten salts and the metallic fin (see Fig. S1 in Supplementary Information), and the energy conversion efficiency was positively dependent on the temperature difference between the hot side and the cold side. Once the light is turned off, the stored thermal energy can be released via the liquid-to-solid phase change at near-constant temperatures, thus enabling the thermoelectric generator to continue electrical power generation.

**Figure 2:**
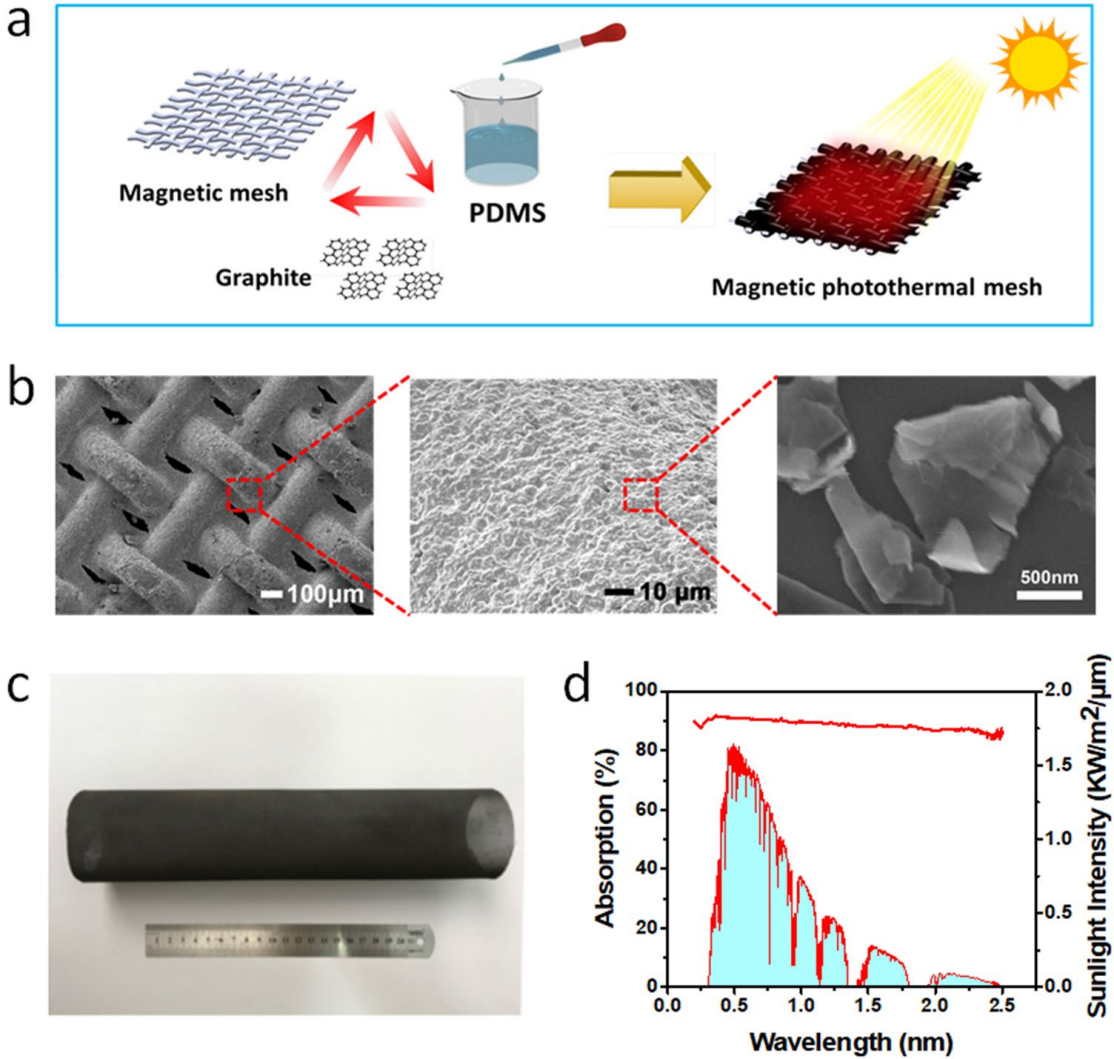
Preparation and characterization of MSCM. (**a**) Schematic for preparation of magnetically-response solar–thermal conversion mesh (MSCM) through a dip-coating method. (**b**) SEM images of MSCM under different magnifications showing its porous structure and enhanced surface roughness with graphite-PDMS coating. (**c**) An optical photograph of MSCM with a large size. (**d**) A UV–Vis–NIR absorption spectrum of MSCM and its comparison to solar irradiance.

As a solar–thermal converter, the MSCM played an important role in the whole charging process. In this work, we adopted a dip-coating method to deposit a photothermal conversion layer onto the surface of a magnetic iron mesh (Fig. 2a). The coating solution was composed of graphite, PDMS and hexane. Owing to its inherent porous structure, chemical stability and strong magnetic response, the low-cost commercial magnetic iron mesh (80-mesh) that has the potential for large-scale fabrication was employed as the supporting material. Considering its high solar absorption, good thermal and chemical stability, graphite with an average size of ~ 0.5 μm was used as the solar-absorbing material in the experiment. PDMS was used as the binder due to its excellent chemical and good thermal stability at high temperatures.

The SEM images in Fig. 2b show that the graphite-PDMS composite coating is uniformly deposited onto the surface of the iron meshes. The entire surface of the coated magnetic iron mesh was wrapped by a layer of graphite-PDMS composite. The average size of graphite particles was about 0.5 μm. The graphite coating layer not only enhanced solar absorptance but also increased the surface roughness. The pore size of the MSCM was about 50 μm, which allows for its penetration through the melted molten salts under magnetic attraction. The untreated pristine iron mesh has a water contact angle of 95°. After depositing the photothermal coating layer, the water contact angle on the iron mesh increases to 154° (see Fig. S2 in Supplementary Information). The hydrophobic surface effectively prevents the direct contact between corrosive melted liquid molten salts and the iron mesh, thus the MSCM has stronger resistance to corrosion than the untreated iron mesh. In the meanwhile, the hydrophobicity of the MSCM helps reduce motion resistance when it travels within the melted molten salts, thereby facilitating the rapid charging process.

Figure 2c presents that a large-sized MSCM can be easily prepared by the facile dip-coating method. As shown by the photograph in Fig. 2c, the flexibility of the iron mesh is not affected by the coating of the photothermal conversion layer and the fabricated MSCM can be rolled. The mechanical flexibility and rollability would facilitate the storage and transportation of MSCM. After coating with the graphite-PDMS composite, the iron mesh turned black. The obtained MSCM was cut into different sizes and shapes for further solar–thermal–electric conversion experiments. As shown by the UV–Vis–NIR absorption spectrum in Fig. 2d, after coating the graphite-PDMS layer the MSCM has a broadband absorption from 0.25 to 2.5 μm, which fully covers the solar irradiance spectrum. The MSCM has shown more than 90% absorption in the UV–Vis range, and the solar absorption of the MSCM is more than 85% in the near-IR region (1.0–2.5 μm).

**Figure 3:**
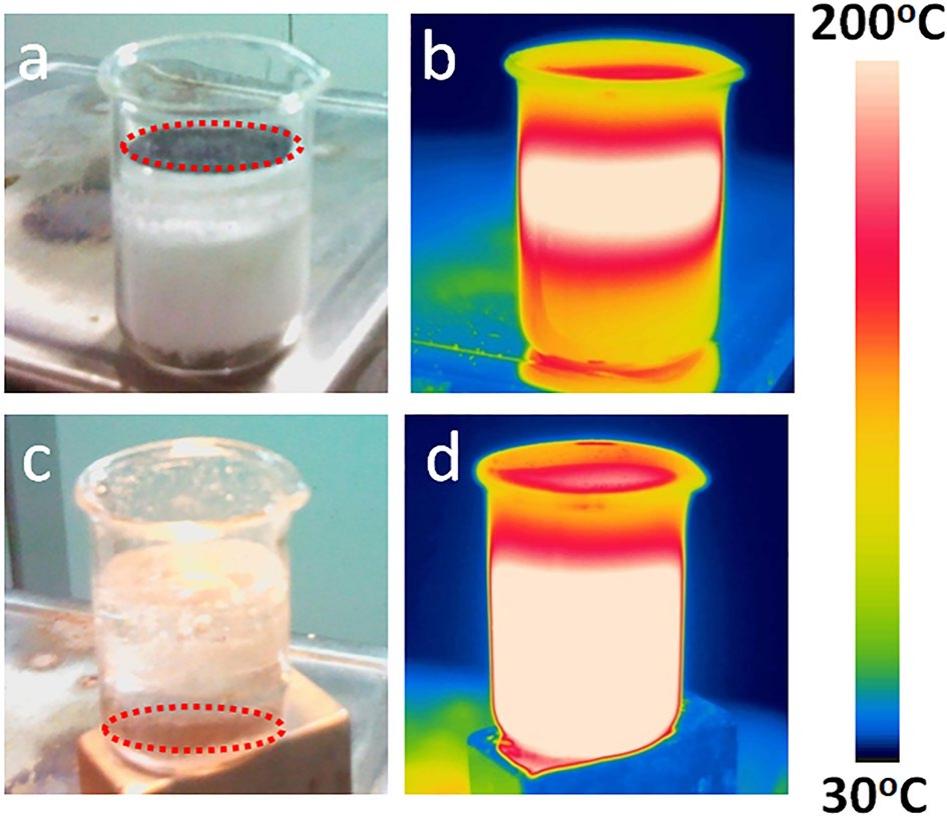
Photographs and IR images of charged molten salts under (**a**, **b**) fixed and (**c**, **d**) movable charging mode. The red circle highlights the position of the MSCM.

High-temperature phase change thermal storage materials were used to store the converted thermal energy by the MSCM as phase change enthalpy and sensible heat, which together provide the energy to drive the thermoelectric conversion. In the experiments, the high-temperature molten salts were chosen as the thermal storage materials. The molten salts used in the experiment are known as the commercial solar salts, which consist of 60 wt% NaNO_3_ and 40 wt% KNO_3_ and are widely used in many concentrating solar power plants such as Solar Two, Andsol1, Andsol2, Andsol3, etc^42,43^. Such nitrate-based solar salts have many attractive features such as low cost, high heat capacity and low operative pressure. The phase change behaviors of the solar salts were measured by a differential scanning calorimeter (DSC). The DSC measurement shows that the solar salts have an onset melting temperature of 210 °C and a phase change enthalpy of 121 J/g (see Fig. S3 in Supplementary Information). Due to the high density and the large surface tension of the melted solar salts, the MSCM floats at the air–liquid salt (Fig. 3). In contrast, when a magnet was placed at the bottom of the container, the MSCM was attracted downwards and it moved together with the receding solid–liquid melting interface. Under this circumstance, once the solid molten salts on the top is melted, the MSCM immediately passes through the liquid phase and pushes the advancement of the solid–liquid interface. Through this movable charging, the solar–thermal charging rate can be doubled if compared to the fixed charging process^41^.

**Figure 4:**
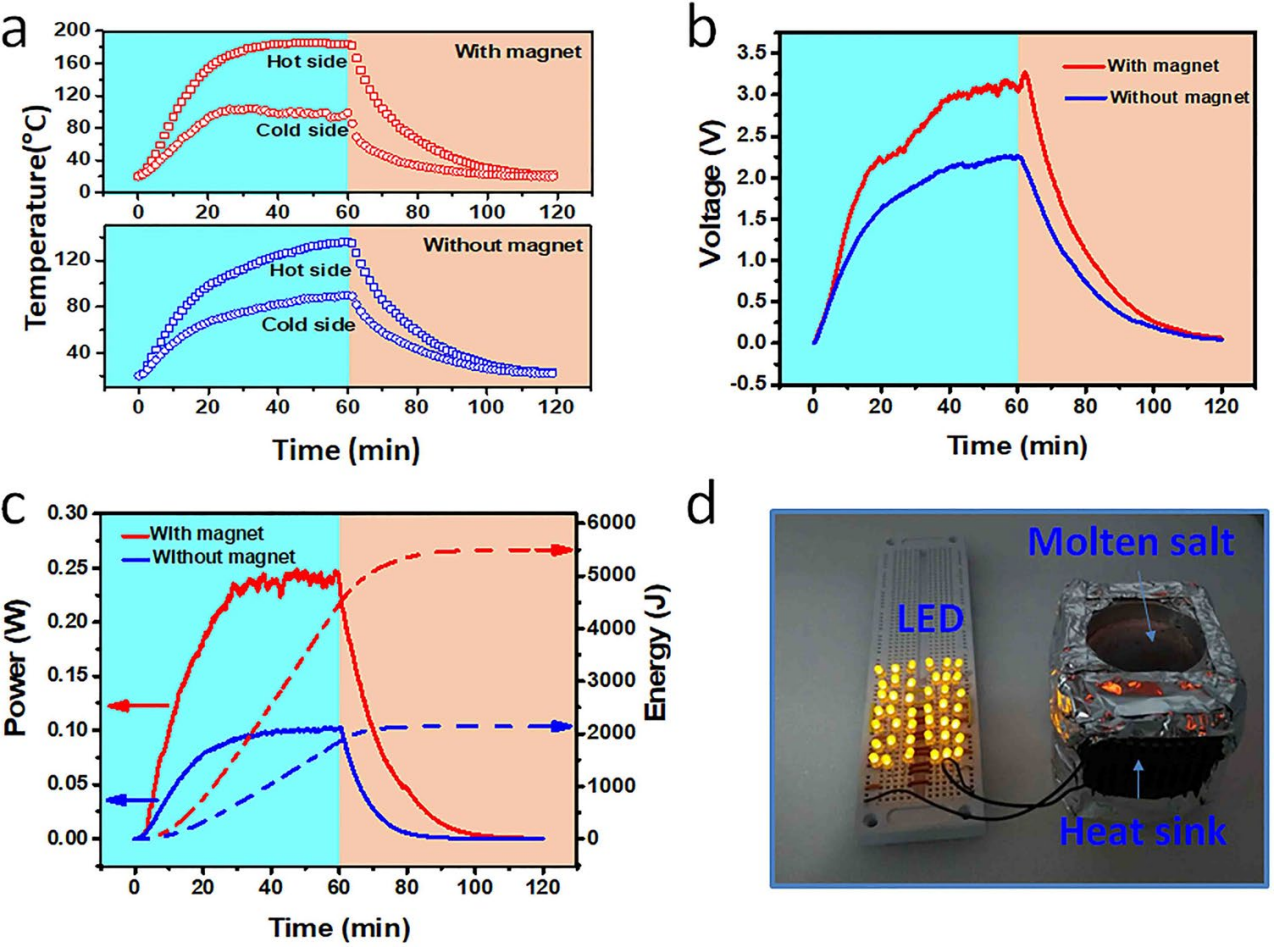
Solar–thermal–electric conversion performance. (**a**) Comparison of temperature variation on both sides of the thermoelectric generator under fixed (bottom) and movable (top) charging mode. (**b**) Comparison of output voltage under fixed and movable charging mode. (**c**) Comparison of generated electricity under fixed and movable charging mode. (**d**) A photograph showing that under the movable charging mode the electrical energy converted from the harvested solar–thermal energy within molten salts is able to light up 42 orange-colored LEDs in parallel.

By connecting the charged system to a thermoelectric generator, we showed that the harvested solar–thermal energy within high-temperature molten salts could produce sufficient electricity to light up multiple light-emitting diodes (LEDs). Compared to the system charged with conventional fixed solar heating, the movable charging led to increased voltage output and prolonged duration of electricity generation after removal of solar illumination. Two thermocouples were used to monitor the temperature variations on the hot side and the cold side of the thermoelectric chip, respectively. In order to reduce the thermal resistance, thermal grease was used between the thermoelectric chip and the aluminum container. Figure 4 presents the performance of the solar energy harvesting system in a movable and fixed charging mode. Under the attraction from the magnet, the solar–thermal storage subsystem was charged under a movable charging mode. Under a solar power density of 30 kW m^−2^, the temperature of the hot side increased to 200 °C within the first 20 min, and then kept constant. In the meantime, the cold side temperature was stabilized at ~ 100 °C as shown by top temperature profile in Fig. 4a, indicating that the maximum temperature difference was 100 °C. Whereas, without the magnet, the solar–thermal storage subsystem was charged under a fixed charging mode. In this case, although the MSCM can efficiently convert incident solar energy into heat the coverted heat is difficult to transfer downward due to the low thermal conductivity of the molten salts. As shown by the bottom temperature profile in Fig. 4a, under the fixed charging mode the hot side temperature was only 120 °C and the cold side temperature of the thermoelectric generator was about 60 °C. The corresponding temperature difference decreased to 60 °C.

The output voltage of the thermoelectric generator is dependent on the temperature difference between the two sides. Figure 4b presents the output voltage of the solar–thermal–electric conversion system under two different charging modes. Under the fixed charging mode, the maximum output voltage is about ~ 2.0 V. Once the light was turned off, the output voltage quickly dropped. By contrast, under the movable charging mode, the maximum output voltage can be increased to ~ 3.3 V due to the larger temperature difference between the two sides of the thermoelectric chip. More importantly, the output voltage could maintain above 1.5 V for 15 min after the light was turned off, which is longer than the lasting duration achieved when the system was charged under the fixed charging mode (8 min). These results show that the amount of solar–thermal energy stored in the molten salts under the movable charging mode is more than that stored by the system under the fixed charging mode, which leads to increased maximum output voltage (165%) and duration of the output (187.5%).

To estimate the overall solar–thermal–electric energy conversion efficiency, the thermoelectric generator was connected to an external resistor with a resistance (*R*) of 5 Ω. The output power can be calculated by the following equation:

1 $$q = \frac{{U^{2} }}{R}$$

where *U* is the output voltage of the thermoelectric generator. Figure 4c presents the output power of the solar–thermal–electric conversion system under two different charging modes. Under the fixed charging mode, the output power is less than ~ 0.1 W after solar irradiation for 30 min. Under the movable charging mode, the output power can reach ~ 0.25 W, which is more than two times of the one achieved under conventional fixed charging mode. The generated electrical energ *P* can be described by:

2 $$P = \mathop \smallint \limits_{0}^{t} qdt$$

where *t* is the total duration for electrical power generation (120 min). Through integrating the output voltage profile with time, the amount of generated electrical energy was calculated to be 5500 J and 2100 J for the system charged under the movable and the fixed charging mode, respectively (Fig. 4c). The overall solar–thermal–electric conversion efficiency $\eta$ can be determined by:

3 $$\eta = \frac{P}{{Aq_{{\text{s}}} t^{\prime } + q_{{\text{c}}} t}}$$

where *A* is the surface area of the MSCM (19.6 cm^2^), $q_{{\text{s}}}$ is the solar power density (30 kW m^−2^), $t^{\prime }$ is the charging time (60 min), and $q_{{\text{c}}}$ is the power consumed by the cold side of the thermoelectric chip (0.45 W). Based on Eq. (3), the solar–thermal–electric energy conversion efficiency achieved under the movable and fixed charging mode was calculated to be 2.56% and 0.97%, respectively. Considering that the aluminum container has four sidewalls and one bottom surface, if they were all connected to thermoelectric chips the overall solar–thermal–electric energy conversion efficiency might be further increased. In addition, the fan cooling may be not necessary in practical applications. For example, the cold side of the thermoelectrical generator can be exposed to cold flowing air or is immersed within cold water. In this case, the large temperature difference between the hot side and cold side is achieved without consumption of electricity, thereby further imporving the energy conversion efficiency.

Unlike low-temperature solar–thermal storage systems that can only generate low output voltage (~ 0.2 V)^38^, the high-temperature molten salt-based storage system has large output voltage and large electrical energy output, thus it can provide sufficient power to drive electronic devices. Figure 4d presents that the electrical power converted from harvested solar–thermal energy can simultaneously light up more than 40 organe-colored LEDs that are connected in parallel. It can be expected that the solar–thermal–electric systems can harvest solar energy during the daytime, and released the stored energy at night to provide both general light illumination and domestic heating. The whole solar–thermal–electric conversion system is made of cheap commercially available materials and all the materials are reusable, which would make it promising for practical widespread applications.

## Conclusions

In summary, this work demonstrated an approach for improving the performance of solar–thermal–electric conversion systems through introducing movable charging of high-temperature molten salts in the solar–thermal storage subsystem. Magnetically-responsive solar–thermal conversion meshes were driven by external magnet field to rapidly advance the solid–liquid melting interface in the molten salts, thereby quickly storing solar–thermal energy. Compared to the fixed charging mode, the movable charging mode increased both the maximum output voltage and the duration of electrical output. The overall efficiency of solar–thermal–electric conversion system reached 2.56%, and the converted electricity was able to simultaneouslylight up more than 40 orange-colored LEDs. It is expected that this work offers a promising strategy for improving the harvesting and conversion performance of solar–thermal energy, thus helps expand the application of solar–thermal technologies.

## Methods

### Materials

Iron meshes (80-mesh) were obtained from Shanghai Hongxiang Co., Ltd. Graphite particles were ordered from Nanjing XFNANO Materials Tech Co., Ltd. Polydimethylsiloxane (PDMS, Sylgard 184) was purchased from Dow Corning Corporation. Potassium nitrate, sodium nitrate, acetone, ethanol and n-hexane were obtained from Aladdin Reagent (Shanghai, China). The thermoelectric chip (TEG1-127–1.0–1.3) was purchased from Harbin Hajing Electronic Co., Ltd.

### Preparation of magnetically-responsive solar–thermal conversion mesh

The magnetically-responsive solar–thermal conversion meshes were prepared by a dip-coating method by following the published protocol^41^. The pristine iron mesh was washed by ethanol and acetone under sonication. The coating solution was prepared by mixing 1 g of graphite, 1 g of PDMS (Part A), 0.1 g of curing agent (Part B) and 20 g of n-hexane. The solution was magnetically stirred at 25 °C for 2 h. The as-cleaned iron meshes were immersed in the coating solution, and then was dried within an oven at 60 °C for 24 h.

### Fabrication of solar–thermal–electric conversion system

The solar–thermal–electric conversion system is composed of a solar–thermal harvesting subsystem and a thermoelectric conversion subsystem. The solar–thermal harvesting subsystem is integrated within an aluminum container with a size of 50 mm × 50 mm × 60 mm. A thermoelectric chip (30 mm × 30 mm × 3 mm) was attached to one side of the device. The cold side of the thermoelectric generator was connected to a metallic fin (35 mm × 35 mm × 10 mm) that was cooled by a fan. 170 g of molten salts were loaded within the container, and a magnetically-responsive solar–thermal conversion mesh with a diameter of 5 cm was placed on top of the molten salts. A magnet is placed at the bottom of the container to attract the downward movement of the photothermal mesh.

### Measurement and characterization

The microstructure of graphite and the movable photothermal meshes was observed by a field emission scanning electron microscope (SEM, Sirion 2000, FEI). The absorption spectrum of the movable photothermal meshes was measured by a UV–Vis–NIR Spectrometer (PerkinElmer, Lambda 750S). The fusion enthalpy of the molten salts was measured by a differential scan calorimeter (Netzsch, 204F1). A solar simulator (TRM-PD, Jinzhou Sunshine Technology Co., Ltd.) was used as the solar light source to generate the large-flux solar illumination (30 kW/m^2^). Output voltage and temperature distribution of the device were recorded by a multichannel data acquisition system (Agilent 34972A).

## Supplementary information

Supplementary Information 1.
