# Supplementary material for: High-efficiency solar thermoelectric conversion enabled by movable charging of molten salts
Source: Sci Rep. 2020 Nov 24;10:20500. doi: 10.1038/s41598-020-77442-y (PMC7687880; doi:10.1038/s41598-020-77442-y)
Supplement: Supplementary file 1 — Supplementary Information 1. [file 41598_2020_77442_MOESM1_ESM.docx]

**Supplementary Information**

**High-efficiency solar thermoelectric conversion enabled by movable charging of molten salts**

Chao Chang,^1,*^ Zongyu Wang,^1^ Benwei Fu^2^ and Yulong Ji^1^

^1^Institute of Marine Engineering and Thermal Science, Marine Engineering College, Dalian Maritime University, Dalian 116026, P. R. China

^2^State Key Laboratory of Metal Matrix Composites, School of Materials Science and Engineering, Shanghai Jiao Tong University, Shanghai 200240, China

^*^Email: [chang3223426@126.com](mailto:chang3223426@126.com)


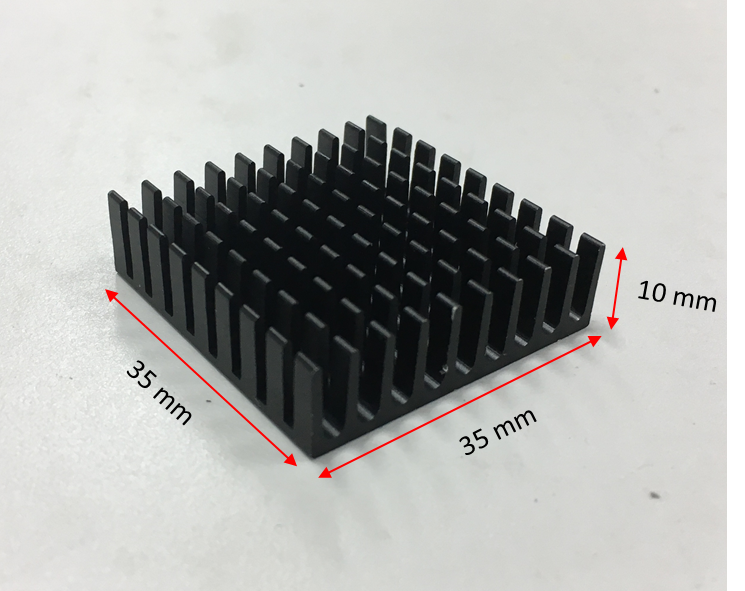


**Figure S1**. Optical image of the metallic fin.


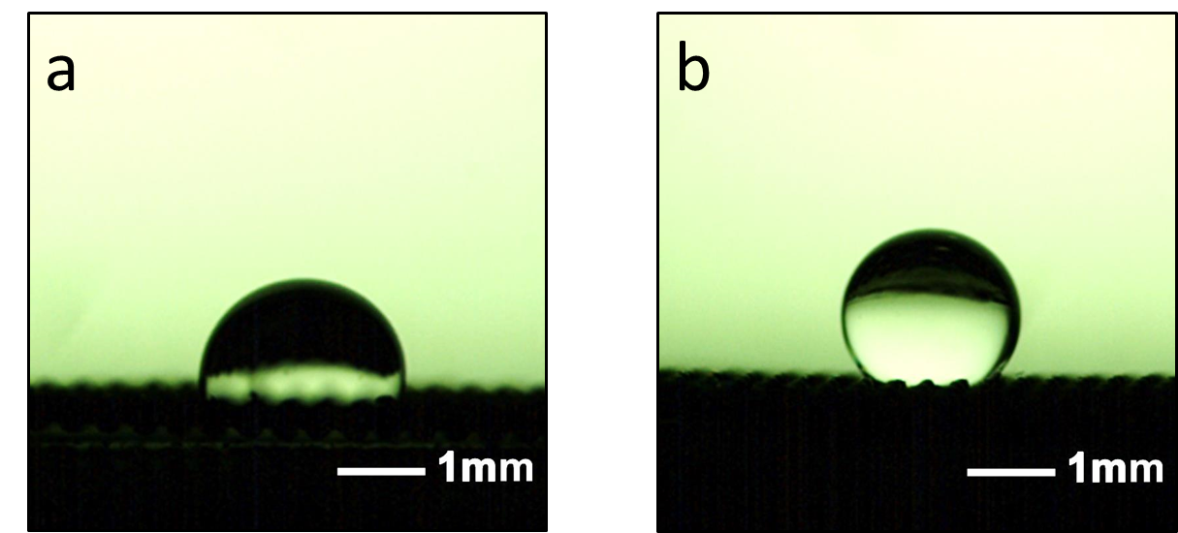


**Figure S2**. Water contact angle measurements for (a) the pristine iron mesh and (b) the treated iron mesh.


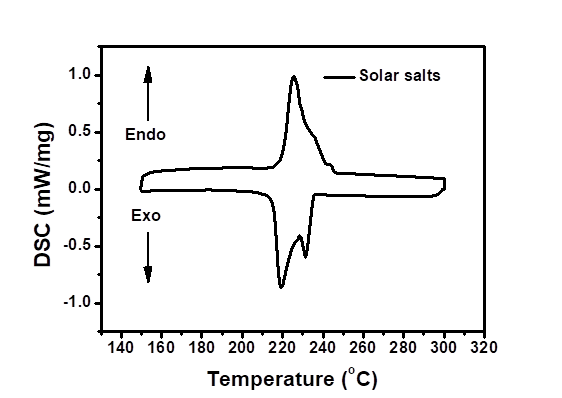


**Figure S3.** DSC curve of solar salts (60 wt% NaNO_3_, 40 wt% KNO_3_) showing an onset melting temperature of 210 ^o^C.
